# Early vs. Late Chemoradiation Therapy and the Postoperative Interval to Adjuvant Therapy Do Not Correspond to Local Recurrence in Resected Pancreatic Cancer

**DOI:** 10.4172/2165-7092.1000151

**Published:** 2015-04-02

**Authors:** Ajay A Patel, Sairaman Nagarajan, Eli D Scher, Caitlin AB Schonewolf, Sairam Balasubramanian, Elizabeth Poplin, Rebecca Moss, David August, Darren Carpizo, Laleh Melstrom, Salma K Jabbour

**Affiliations:** 1 Department of Radiation Oncology, Rutgers Cancer Institute of New Jersey, State University of New Jersey, USA; 2 Center on Genomics, Massachusetts General Hospital, Harvard Medical School, Boston MA, USA; 3 Rowan University School of Osteopathic Medicine, Stratford NJ, USA; 4 Department of Radiation Oncology, University of Pennsylvania, Philadelphia PA, USA; 5 Department of Medicine, Division of Medical Oncology, Cancer Institute of New Jersey; 6 Department of Surgery, Division of Surgical Oncology, Rutgers Cancer Institute of New Jersey, Rutgers University, New Brunswick NJ, USA

**Keywords:** Adjuvant therapy, Chemoradiation, Pancreas, Interval, Sequencing, Recurrence, Survival

## Abstract

**Objective:**

Standard postoperative therapy for pancreatic cancer consists of both chemotherapy alone and chemoradiation. We sought to investigate whether the sequence of chemotherapy and chemoradiation and overall time to initiation of adjuvant therapy would impact local vs. distant recurrence.

**Methods:**

After Institutional Review Board approval, resected pancreas cancer patient charts were evaluated for medical background, surgical, pathological, chemoradiation (CRT), and follow-up. Local recurrence (LR) was defined as failures occurring in the postoperative bed and regional lymph nodes. Early vs. late CRT was defined by whether CRT was given early (within 1–2 cycles of adjuvant chemotherapy) or late in the course of adjuvant chemotherapy (after the 3rd cycle of chemotherapy). The postoperative interval variance was compared to LR factors such as progression-free survival (PFS) and overall survival (OS).

**Results:**

Of the 34 eligible patients, 47% (n=16) underwent early CRT and 41% (n=14) underwent late CRT. 12% (n=14) did not undergo any induction chemotherapy. At median follow-up of 22 months, 53% (n=18) had metastases, 24% (n=8) had LR, and 24% (n=8) were disease free. Kaplan-Meier curves revealed that early vs. late CRT did not appear to significantly impact OS (p=0.63), PFS (p=0.085) or LR (p=0.19). Postoperative interval did not affect PFS (p=0.42) or OS (p=0.93).

**Conclusions:**

Early vs. late CRT and the time to initiation of adjuvant therapy were not significantly associated with LR in patients with resected pancreatic cancer. Future prospective studies are required to determine if sequencing of chemotherapy, CRT, or the postoperative interval impact survival and patterns of recurrence.

## Introduction

Pancreatic cancer is the fourth leading cause of cancer-related death in the United States, and roughly 20% of the patients diagnosed are able to undergo potentially curative surgical resection. Even among those with resected pancreatic cancer, the 5-year survival rate hovers under 20%, and drops to about 10% for those with even one positive lymph node [1,2]. Thus, adjuvant therapy is typically employed to potentially prevent recurrence and improve the rate of overall survival (OS).

Multiple studies have evaluated adjuvant chemoradiation (CRT) after radical surgery for pancreatic cancer [3,4]. The Gastrointestinal Tumor Study Group compared surgery alone to surgery followed by 5-fluorouracil (5-FU)-based CRT and maintenance 5-FU, which demonstrated an improvement in OS and lead to the adoption of CRT as the preferred immediate post-surgical treatment [5]. The RTOG 9704 trial compared pre- and post-CRT 5-FU to pre- and post-CRT gemcitabine in the postoperative setting for resected pancreatic adenocarcinoma, and demonstrated no difference in survival between these two regimens. In that trial, one cycle of chemotherapy, then CRT, then four cycles post-CRT was delivered [6].

Due to the preponderance of distant metastasis (DM), a delay in adjuvant CRT has been endorsed in favor of early chemotherapy to reduce the risk of developing DM during CRT [7]. The current RTOG 0848 study randomizes patients to two chemotherapy regimens (gemcitabine +/− erlotinib) for five cycles followed by restaging imaging, and a second randomization to an additional cycle of chemotherapy alone or one cycle of chemotherapy followed by CRT. It is unclear if this empiric delay in CRT impacts local or distant control rates or survival, as the optimal timing of CRT remains a matter of debate even for other cancer types [8,9]. Owing to the historically >70% rate of distant metastatic failures, adjuvant CRT was deferred over time to be delivered at later stages in the patients’ postoperative course in favor of earlier chemotherapy. However, there has been little research to determine if delaying CRT impacts outcomes.

We sought to analyze a cohort of resected pancreatic cancer patients treated at our institution with the goal of investigating whether

1. Timing of CRT (early vs. late) in relation to adjuvant chemotherapy, and
2. Time to initiation to adjuvant therapy would impact the pattern of recurrence or survival.

## Methods and Materials

### Data collection

After Institutional Review Board approval, data were retrospectively collected from radiation oncology records in the Aria^®^ electronic medical record system (Varian Medical Systems, Palo Alto, CA) exclusively for patients who underwent pancreatic resection for pancreatic adenocarcinoma and adjuvant radiation therapy. These patient records were cross-referenced with respective medical oncology charts to determine the prescription of adjuvant chemotherapy. The standard record evaluation for each patient included a complete medical history, operative and pathological reports, adjuvant chemotherapy and radiation therapy records, and physician post-treatment records including radiological examinations. Timing and first site of failure were also assessed.

Extracted and systematized demographical variables included: surgical procedure, tumor and nodal stage, margin status, number of lymph nodes collected/positive, histology, differentiation, cycles of adjuvant chemotherapy (pre- and post-RT) and radiation therapy, dosage fractionation (Gy), and concurrent chemotherapy. Dates of surgery and schedules of adjuvant therapy regimens were then used to calculate intervals between surgery and both adjuvant chemotherapy and CRT, duration of CRT, and follow-up time from surgery to recurrence.

The timing of CRT was categorized into groups of early vs. late; the early group received two cycles or fewer of early adjuvant chemotherapy before CRT, while the late group received CRT after at least three cycles of adjuvant chemotherapy. Recurrences were defined based on the first radiographic and clinical interpretation of the return of disease. LR was defined as occurring within the radiation field in the region of the primary tumor or regional lymph nodes (peripancreatic, pancreaticoduodenal, celiac, para-aortic, porta hepatic). Metastatic was defined as recurrence outside the radiation field. Recurrence was coded as local or metastatic based on first site of recurrence.

### Chemotherapy Delivery

The majority of patients (88%) received postoperative chemotherapy composed of gemcitabine at 1000 mg/m2 before radiation therapy. Computed tomography (CT) restaging was performed after chemotherapy to ensure no progression of disease was present. When patients were deemed fit for CRT and began treatment, almost all (91%) underwent concurrent chemotherapy with gemcitabine 400 mg/m2 weekly, capecitabine 825 mg/m2 BID, or 5-FU 225–250 mg/m2 continuous infusion. At the completion of CRT, 59% of all patients underwent further post-RT chemotherapy, with all receiving gemcitabine. For the remaining 61% of patients, treatment concluded with the completion of RT.

### Radiation Therapy Delivery

The clinical target volume (CTV) included the preoperative tumor volume via preoperative image fusion, incorporation of any clips left by the surgeon with the intention of delineating areas at risk, and regional lymph nodes; the latter included peri-pancreatic, pancreaticoduodenal, celiac, para-aortic, and portahepatic lymph nodes. Anastomotic sites including pancreaticoduodenal anastomosis and choledochojejunostomy and operative bed were also included for head of pancreas tumors.

### Statistical Analysis

All analyses were conducted using SAS v. 9.3 (SAS Institute, Cary, NC) and the statistical level of significance was 0.05 unless otherwise specified. Bivariate analyses for pairwise associations between baseline characteristics (contingency tables) were conducted using Chi-square tests of significance and their corresponding p-values are reported. Comparisons between patterns of recurrence groups were conducted using the nonparametric Kruskal-Wallis test for continuous variables or Fisher’s exact test for discrete variables. Univariate logistic regression was used to determine which factors were significantly associated with LR (data not shown). Unadjusted Kaplan-Meier curves for product limit survival estimates were computed with censored sample observations. These curves were for OS, progression-free survival (PFS) and local recurrence (LR) from the timing of initiation of CRT. Log rank test and Fleming statistics comparing survival strata for overall CRT initiation are reported.

## Results

### Baseline Patient Characteristics

In total, 34 patients treated between July 2004 and October 2011 was eligible for this investigation.

Patient characteristics are listed in Table 1. Median follow-up time was 22 months.

The majority of patients underwent pancreaticoduododectomy (76%) and was found to have T3 disease with a negative margin status. Most (88%) were also treated first with induction chemotherapy, followed by CRT and then further chemotherapy. Nearly half (47%) of patients underwent between 1–2 cycles of induction chemotherapy (early CRT group), while 41% underwent 3–7 cycles of induction chemotherapy before CRT (late CRT group), and 12% did not undergo any induction chemotherapy. Almost all (97%) patients received concurrent chemotherapy alongside radiation therapy.

### Survival

**Figure 1:**
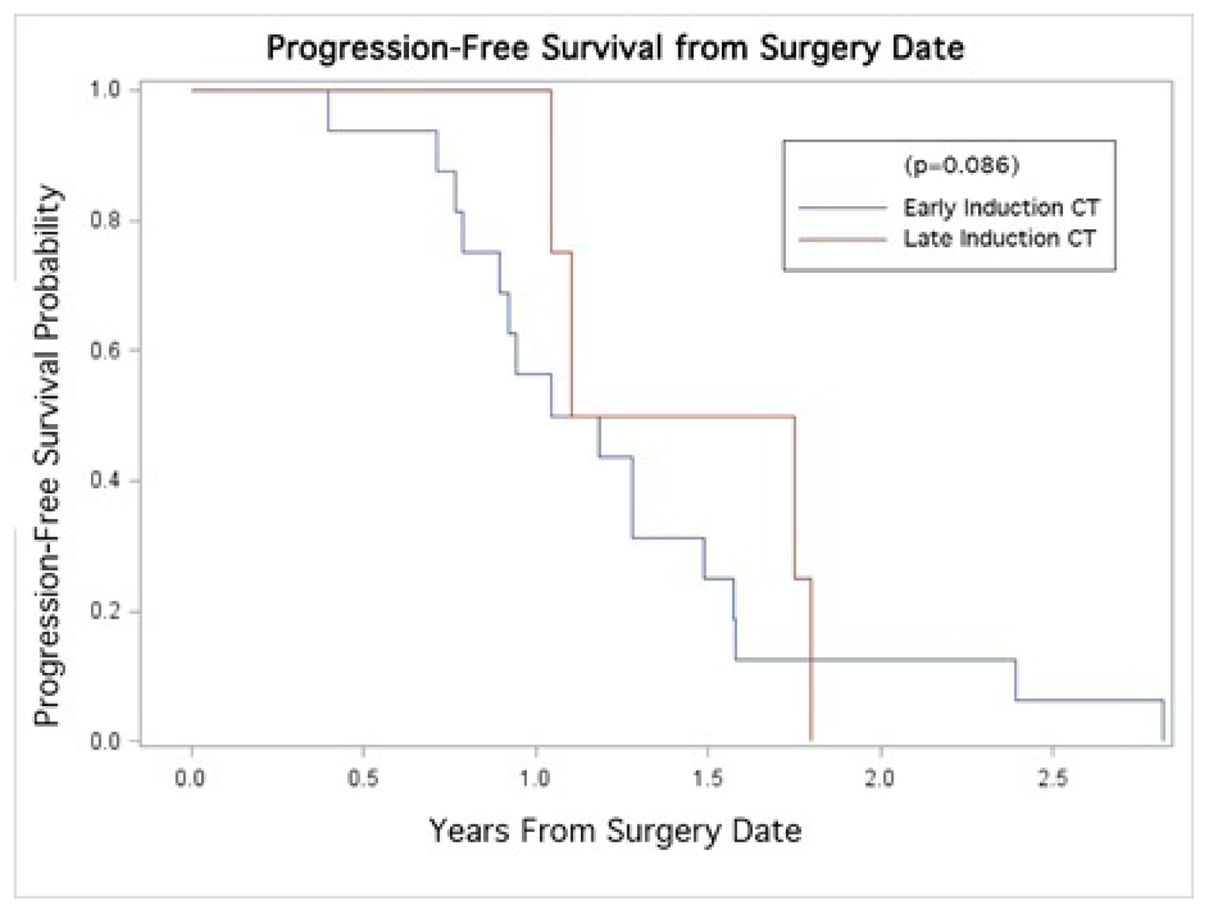
Kaplan-Meier curve for Progression-Free survival stratified by time to adjuvant therapy (early/late adjuvant CT).

**Figure 2:**
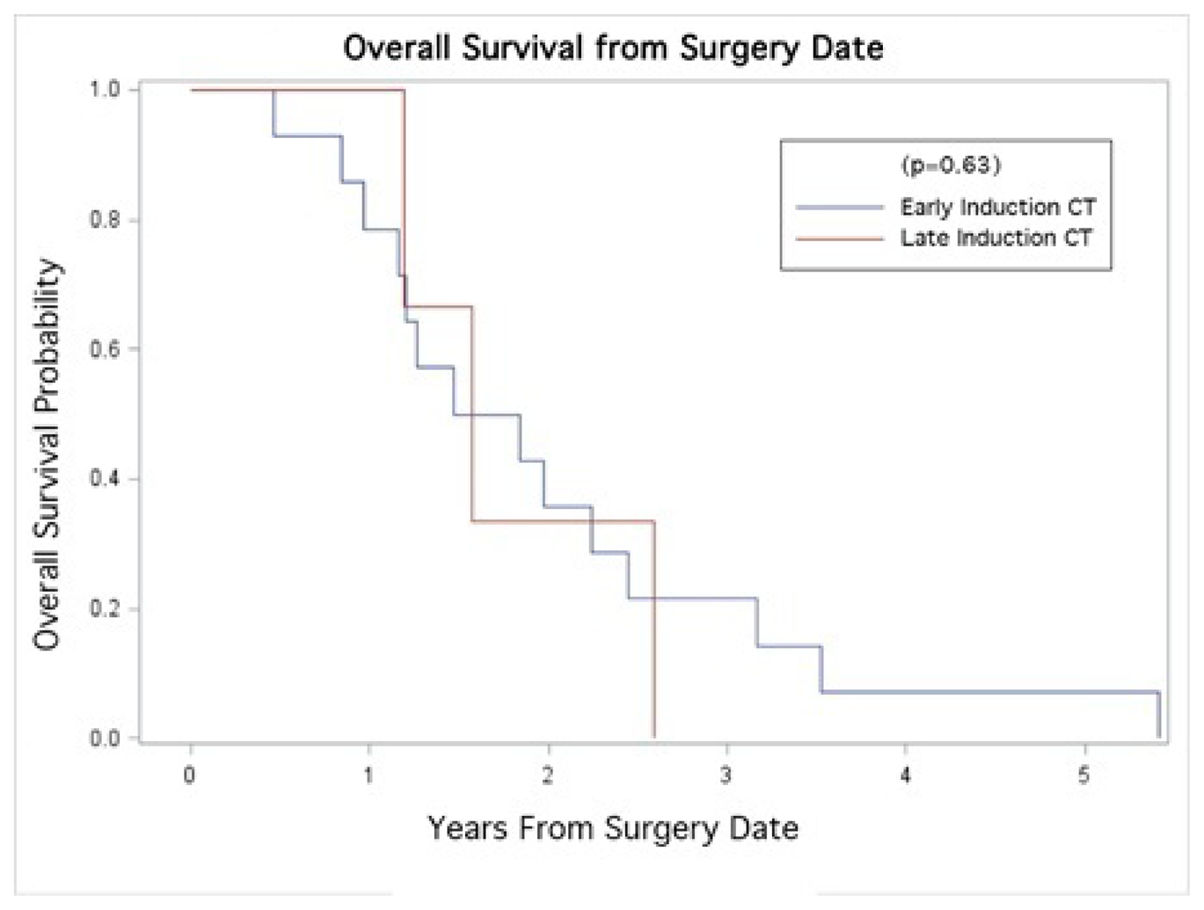
Kaplan-Meier curve for Overall survival stratified by time to adjuvant therapy (early/late adjuvant CT).

**Figure 3:**
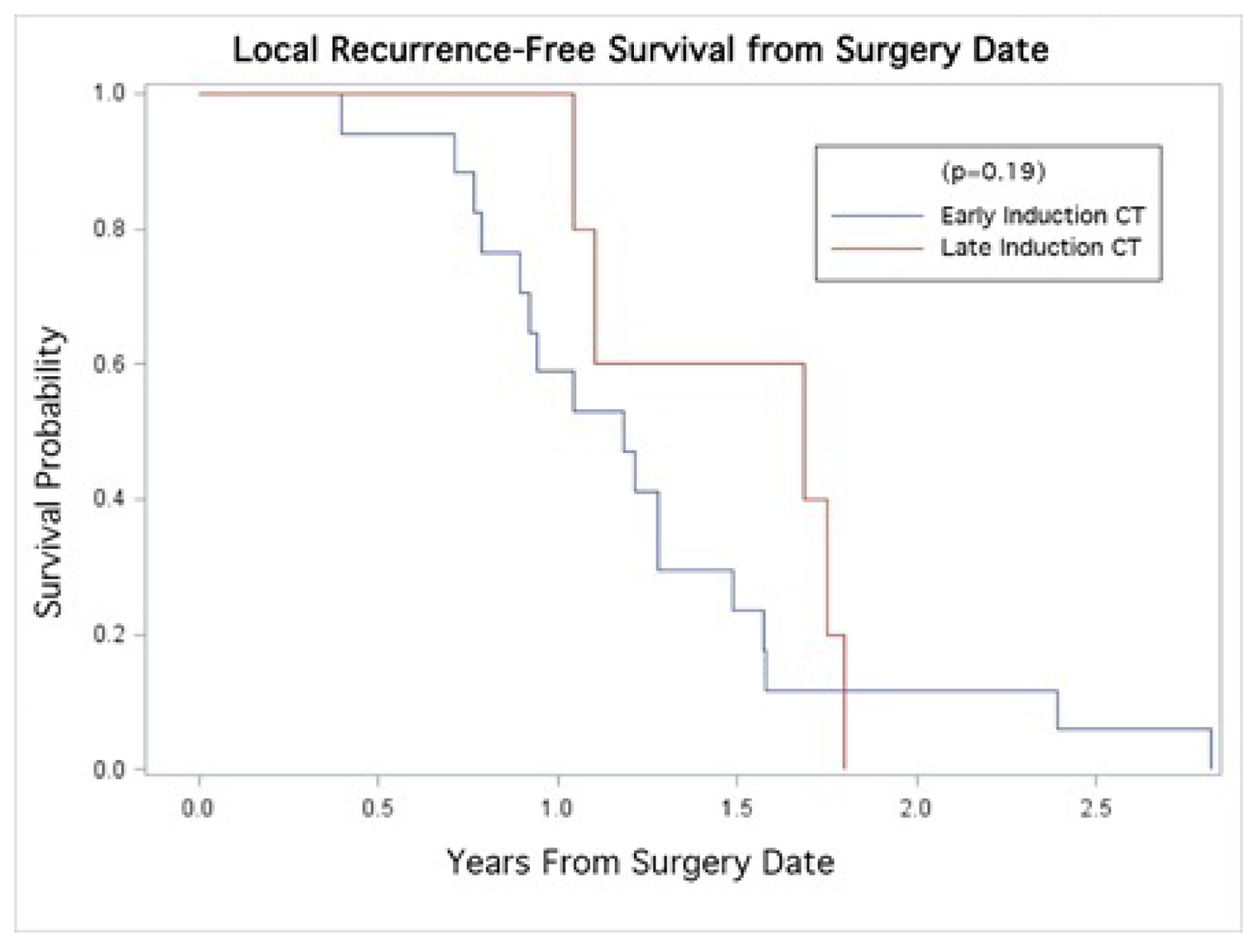
Kaplan-Meier curve for Local Recurrence-Free survival stratified by time to adjuvant therapy (early/late adjuvant CT).

The median PFS was 1.12 years. Kaplan-Meier curves revealed no statistically significant differences in PFS across groups of early and late CRT (p=0.085; Figure 1). The median OS and survival time to LR were 1.52 years and 1.48 years, respectively. Similarly, no significant differences were found in OS (p=0.63; Figure 2), or LR (p=0.19; Figure 3) between early and late CRT groups.

Postoperative details prior to adjuvant therapy and further CRT regimen details are demonstrated in Table 2.

### Patterns of failure

A total of 24% of patients were disease-free (n=8). Of these, 100% (n=8) of disease-free patients underwent induction chemotherapy and 50% (n=4) underwent post-radiation chemotherapy. 63% (n=5) of these patients were in the early CRT group, while the remaining were in the late CRT group.

Locally recurrent disease was found in 24% (n=8) of patients. A total of 53% of patients (n=18) were diagnosed with metastatic disease, which included: liver (n=11), lung (n=3), axilla (n=1), and peritoneum (n=3).

### Timing of CRT and failure

The timing of CRT (early vs. late) had no significant impact on OS (p=0.70). There was also no significant difference in the incidences of either distant or local recurrences between the early and late CRT groups (p=0.36).

### Time to initiation of adjuvant therapy

An analysis of variance (ANOVA) for the mean postoperative interval (number of days) to adjuvant therapy did not show significant differences between groups of: no recurrence, local recurrence, and metastatic recurrence (p=0.073). The mean postoperative intervals for each group were: 62.1 (no recurrence), 82.9 (local), and 46.4 (metastatic) days. Similar analyses of the three groups did not show significant differences for the mean duration of radiotherapy (p=0.42) or days from surgery to recurrence (p=0.80).

Univariate Kaplan-Meier product limit survival estimates showed that days between surgery and initiation of treatment and margin status were not independent predictors of PFS (p=0.42, p=0.71, respectively) or OS (p=0.93, p=0.08, respectively).

## Discussion

The delivery of adjuvant therapy – even in those with resected T1N0 tumors – has largely been established as an effective means of modest improvements in both local control and survival, which are predominantly resultant of adjuvant RT or chemotherapy, respectively [10–14]. Although post-operative therapy is typically started shortly after surgery for most tumors, with pancreatic cancer, there is often a delay to allow for recovery from the high-morbidity operation [15]. Studies have shown, though, that when treated with adjuvant chemotherapy alone, completion of the full course of therapy provided the most significant survival benefit; there was no difference in survival whether postoperative treatment was delayed up to 12 weeks, or started within 8 weeks [15].

In the present study, the mean number of days from the time of surgery to the start of any adjuvant therapy was not found to be an independent predictor of PFS (p=0.42) or OS (p=0.93). These findings are in accordance with available data on adjuvant chemotherapy alone for pancreatic cancer, in which OS was not significantly impacted by changes in timing [15]. Other studies in breast and colorectal cancer have likewise demonstrated similar results for timing of adjuvant chemotherapy [16–20].

Although a phase II trial found that the rate of LR as the first sign of disease progression was lower in the CRT group, there is a lack of randomized data on the ideal timing for employing adjuvant CRT [4,15]. This may be due to the concern for possibly decreased survival rates, which have been proposed as a potential consequence of lowering chemotherapy doses, which is necessary when adding RT to adjuvant regimens [15,16]. In the United States, the current standard is to deliver adjuvant CRT in order to reduce the risk of LR, despite the unclear benefit in OS [13,17]. In the present study, although the timing for the initiation of postoperative therapy varied, the median time from surgery to adjuvant treatment was 6.9 weeks (interquartile range, 5.6–9.4 weeks), which was largely below the eight-week maximum that was examined by the European Organisation for Research and Treatment of Cancer phase II trial, which demonstrated the feasibility of CRT as a beneficial regimen [13].

Our data show no significant change in clinical outcomes from delayed administration of CRT, in terms of either LR (p=0.19) or OS (p=0.63), nor PFS (p=0.086). Others have shown that delaying the initiation of CRT while treating with induction gemcitabine alone allowed for a more complete postoperative recovery, with a subsequent benefit of improved treatment tolerability once CRT was initiated [13]. The importance of this should not be understated, as it has been shown that patients who were able to successfully complete a full course of adjuvant treatment demonstrated a significant survival advantage over those with shortened courses [15]. The ongoing RTOG 0848 trial is a phase III study employing induction chemotherapy alone, followed by CRT toward the end of the prescribed adjuvant chemotherapy treatment, and should help better illustrate the ramifications of adjuvant treatment timing if accrual is completed [13].

The small sample size in the present study was a limitation that likely yielded statistical insignificance that may not necessarily reflect clinical importance. These conclusions drawn from a potentially underpowered data set may not necessarily hold true for all patients across demographics. There were also 29% (n=10) of patients who also had extended delays (>8 weeks) prior to starting adjuvant therapy, which may have distorted the results due to the complications that necessitated those delays. Though our sample size is not optimal for definite conclusions, we do predict that the time prior to adjuvant therapy may not hold much influence over survival in a greater number of cases as well.

## Conclusion

Our results demonstrate that for our patients, neither a delay of CRT nor a shorter interval to starting adjuvant treatment play a significant role in patient outcomes. Whether any potential benefit from early adjuvant treatment can outweigh the advantages of ensuring patient recovery by delaying CRT after a morbid surgical operation remains to be seen. We await the completion and results of RTOG 0848 to inform clinicians of the role and timing of adjuvant chemoradiation after postoperative chemotherapy.

## Figures and Tables

**Table 1:** Baseline Patient Characteristics

Baseline Patient Characteristics	n (%) or n (range)

Gender	

Male	13 (38)
Female	21 (62)

Median Age at Surgery, years	58.5 (40–79)

Type of Pancreatic Surgery	

Pancreaticoduodenectomy	26 (76)
Distal pancreatectomy	7 (21)
Total pancreatectomy	1 (3)

Tumor Stage	

T2	6 (18)
T3	26 (76)
T4	2 (6)

Lymph Nodes Collected	

Median	12.5 (3–21)

Lymph Nodes Positive	

Median	1 (0–14)

Node Stage	

N0	13 (38)
N1	21 (62)

Margin Status	

Negative	21 (62)
Positive	10 (29)
Close	3 (9)

Differentiation	

Poor	8 (24)
Moderate	21 (62)
Well	4 (12)

**Table 2:** Postoperative & CRT Regimen Details

Postoperative and CRT Regimen Details	n (%) or n (range)

Time between Surgery and Adjuvant Therapy, (weeks)	

Median (Interquartile range)	6.9 (5.6–9.4)

Order of Adjuvant Therapy	

CRT Only	2 (6)
CRT? CT	2 (6)
CT?CRT	12 (35)
CT?CRT?CT	18 (53)

Chemotherapy Prior to Chemoradiation Completed	

1 cycle	8 (24)
2 cycles	8 (24)
3 cycles	6 (18)
4 cycles	1 (3)
5 cycles	3 (9)
6 cycles	2 (6)
7 cycles	2 (6)

Radiation Therapy Type	

Intensity-Modulated Radiation Therapy (IMRT)	29 (85)
3D Conformal (3DCRT)	5 (15)

RT Total Dosage	

21.6 Gy	1 (3)
45.0 – 48.6 Gy	7 (21)
50.4 – 54.0 Gy	26 (76)

RT Median Dosage, n/Gy	50.4 [in 28 1.8-Gy fractions]

RT Treatment Elapsed Days, n/days	

Median	38 (16–49)

Type of chemotherapy concurrent with RT	

None received (N/A)	1 (3)
Gemcitabine	7 (21)
Capecitabine	14 (41)
5-FU	12 (35)

Post-RT Chemotherapy	

1 cycle	4 (12)
2 cycles	2 (6)
3 cycles	10 (29)
4 cycles	1 (3)
6 cycles	2 (6)
9 cycles	1 (3)
